# Editorial: Artificial intelligence techniques for personalized educational software

**DOI:** 10.3389/frai.2022.988289

**Published:** 2022-08-10

**Authors:** Christos Troussas, Akrivi Krouska, Katerina Kabassi, Cleo Sgouropoulou, Alexandra I. Cristea

**Affiliations:** ^1^Department of Informatics and Computer Engineering, University of West Attica, Athens, Greece; ^2^Department of Environment, Ionian University, Corfu, Greece; ^3^Department of Computer Science, Durham University, Durham, United Kingdom

**Keywords:** personalized software, human-computer interaction, augmented intelligence, intelligent techniques, artificial intelligence, intelligent tutoring systems, adaptive educational hypermedia systems

Adaptive educational hypermedia systems are technologically advanced applications aiming to provide immediate and tailored instruction or feedback to learners. Their extensive use in education is completely transforming human life, especially during the COVID-19 pandemic. While secure, scalable, and feature-packed learning technology applications are in high demand, the desire for interacting with customized content is ever-growing (Papakostas et al., 2021). Besides adaptive learner interfaces, users are searching for smart learning technology systems that could provide a highly personalized user experience. Artificial intelligence (AI) can face these challenges and implement innovative digital techniques and tools in education. Using intelligence techniques, i.e., machine learning, deep learning, neural networks, reinforcement learning, fuzzy logic, cognitive maps, and genetic algorithms among others, these systems provide innovative features adjusted to human needs and interests (Chen et al., 2021). AI makes educational software more user-centric, helps in implementation of complex tasks and the process of huge data minimizing their execution time, and optimizes the entire system's functionality.

Research on adaptive educational hypermedia systems faces numerous challenges, many of which are related to representing a dynamic physical learning environment computationally and applying it to real-world tutoring problems. In view of this, this Research Topic emphasizes personalized educational software: the methods used, and interdisciplinary research for enabling, supporting, and promoting AI techniques in its development. It aims to regroup and promote high-quality research in the field, creating a forum for challenges and novel advancements in AI in education to be explored. While a great deal of research has been presented in the field (Krouska et al., 2020; Anwar, 2021; Mousavi et al., 2021; Schaldenbrand et al., 2021; Sense et al., 2021; Pelánek, 2022; Rebolledo-Mendez et al., 2022; Zhou et al., 2022), there is a significant room for improvement in this direction. This Research Topic focuses on triggering an exchange of ideas in the field and reinforcing and expanding the network of researchers, academics, and market representatives. It is intended for both experts/researchers and practitioners in the fields of artificial and computational intelligence in intelligent tutoring systems as well as eLearning. The Research Topic aspires to capture the state of the field and articulate an agenda that would push the fields of personalized educational software and learner-interface interaction toward new directions.

The original list of topics for which studies were solicited was as follows:

- Adaptive/personalized strategies and systems in education
- Interactive machine learning in education
- Augmented intelligence
- Intelligent user interfaces in learning technology systems
- Cognitive science in education
- Intelligent techniques (e.g., deep learning, neural networks, reinforcement learning, fuzzy logic, cognitive maps, genetic algorithms, etc.) in education.

A variety of new techniques/strategies had been introduced or revisited, including blockchain in education, instructional design, cognitive training interventions, conversational agents, self-paced instruction, and deep neural networks. The rigor of the reported study had been ironclad and yielded several generalizable results. Moreover, it allowed for a room for the deployment of methods such as observational studies, longitudinal studies, and meta-analyses. From the above list of topics of interest, the articles focused on areas such as adaptive and personalized strategies and systems in education, cognitive science in education, intelligent techniques in education, and intelligent user interfaces in learning technology systems. This is indicating active areas of research where mature enough work has been presented in the field. However, topics such as interactive machine learning in education and augmented intelligence in education received no submissions probably due to lack of mature research studies.

The first article in this Research Topic, authored by Rahardja et al., is titled “*Education Exchange Storage Protocol: Transformation into Decentralized Learning Platform*.” This article suggests that people should migrate from classical centralized storage to innovative decentralized schemes in the education sector. To achieve this purpose, the authors introduce a novel EESP framework coupled with a blockchain smart contract that contributes to making significant changes and improvements compared to the current system.

The second article, authored by Huang et al., is titled “*The Role of Self-Improving Tutoring Systems in Fostering Pre-Service Teacher Self-Regulated Learning*.” The authors review the current state of pertinent research and development of a network-based tutoring system called nBrowser, which is designed to support teacher instructional planning and technology integration.

The third article, authored by Vladisauskas et al., is titled “*A Machine Learning Approach to Personalize Computerized Cognitive Training Interventions*.” The authors propose an initial approach toward cognitive training personalization using machine learning algorithms to try to identify subjects that will (or will not) benefit from a certain protocol of cognitive stimulation.

The fourth article, authored by Casillo et al., is titled “*An Ontology-Based Chatbot to Enhance Experiential Learning in a Cultural Heritage Scenario*.” This article aims to present the results of a study conducted by implementing a chatbot whose responses are fed by a knowledge base related to the Archaeological Urban Park of Naples (PAUN).

The fifth article, authored by Christodoulou and Angeli, is titled “*Adaptive Learning Techniques for Personalized Educational Software in Developing Teachers' Technological Pedagogical Content Knowledge*.” The authors aim to contribute to this line of research by discussing the design and utilization of e-TPCK, a self-paced adaptive electronic learning environment that was developed and used to support the development of student-teachers' technological pedagogical content knowledge (TPCK) in a personalized way during their undergraduate studies.

The sixth article, authored by Tato and Nkambou, is titled “*Infusing Expert Knowledge into a Deep Neural Network Using Attention Mechanism for Personalized Learning Environments*.” The main contribution of this article is to show how an original hybrid deep neural architecture infused with *a priori* expert knowledge through attention mechanism improves its generalization ability. The resulting architecture has shown its effectiveness for learner modeling and personalization in two types of educational software.

The editors of the Research Topic hope that it can be helpful and motivating for its audience and support the research of senior and junior scientists, lecturers, and students. Finally, there is an ever-increasing demand for continuing the research on the above issues, so the Research Topic can offer a fertile ground to this direction.

## Author contributions

All authors listed have made a substantial, direct, and intellectual contribution to the work and approved it for publication.

## Conflict of interest

The authors declare that the research was conducted in the absence of any commercial or financial relationships that could be construed as a potential conflict of interest.

## Publisher's note

All claims expressed in this article are solely those of the authors and do not necessarily represent those of their affiliated organizations, or those of the publisher, the editors and the reviewers. Any product that may be evaluated in this article, or claim that may be made by its manufacturer, is not guaranteed or endorsed by the publisher.
